# Transcriptome analysis reveals the potential lncRNA-mRNA modules involved in genetic male sterility and fertility of Chinese cabbage (brassica rapa L. ssp. pekinensis)

**DOI:** 10.1186/s12870-024-05003-w

**Published:** 2024-04-16

**Authors:** Xiaochun Wei, Xiaoqing Wang, Yanyan Zhao, Weiwei Chen, Ujjal Kumar Nath, Shuangjuan Yang, Henan Su, Zhiyong Wang, Wenjing Zhang, Baoming Tian, Fang Wei, Yuxiang Yuan, Xiaowei Zhang

**Affiliations:** 1 Institute of Vegetables, Henan Academy of Agricultural Sciences, Graduate T & R Base of Zhengzhou University, Zhengzhou, 450002 Henan China; 2 School of Agricultural Sciences, Zhengzhou University, Zhengzhou, 450001 Henan China; 3 Department of Genetics and Plant Breeding, Bangladesh Agricultural University, Mymensingh, 2202 Bangladesh

**Keywords:** Chinese cabbage, Genic male sterility, GO analysis, LncRNA

## Abstract

**Background:**

Long non-coding RNAs (lncRNAs) play a crucial role in regulating gene expression vital for the growth and development of plants. Despite this, the role of lncRNAs in Chinese cabbage (*Brassica rapa* L. ssp. *pekinensis*) pollen development and male fertility remains poorly understood.

**Results:**

In this study, we characterized a recessive genic male sterile mutant (366–2 S), where the delayed degradation of tapetum and the failure of tetrad separation primarily led to the inability to form single microspores, resulting in male sterility. To analyze the role of lncRNAs in pollen development, we conducted a comparative lncRNA sequencing using anthers from the male sterile mutant line (366–2 S) and the wild-type male fertile line (366–2 F). We identified 385 differentially expressed lncRNAs between the 366–2 F and 366–2 S lines, with 172 of them potentially associated with target genes. To further understand the alterations in mRNA expression and explore potential lncRNA-target genes (mRNAs), we performed comparative mRNA transcriptome analysis in the anthers of 366–2 S and 366–2 F at two stages. We identified 1,176 differentially expressed mRNAs. Remarkably, GO analysis revealed significant enrichment in five GO terms, most notably involving mRNAs annotated as pectinesterase and polygalacturonase, which play roles in cell wall degradation. The considerable downregulation of these genes might contribute to the delayed degradation of tapetum in 366–2 S. Furthermore, we identified 15 lncRNA-mRNA modules through Venn diagram analysis. Among them, *MSTRG.9997-BraA04g004630.3 C* (β-1,3-glucanase) is associated with callose degradation and tetrad separation. Additionally, *MSTRG.5212-BraA02g040020.3 C* (pectinesterase) and *MSTRG.13,532-BraA05g030320.3 C* (pectinesterase) are associated with cell wall degradation of the tapetum, indicating that these three candidate lncRNA-mRNA modules potentially regulate pollen development.

**Conclusion:**

This study lays the foundation for understanding the roles of lncRNAs in pollen development and for elucidating their molecular mechanisms in regulating male sterility in Chinese cabbage.

**Supplementary Information:**

The online version contains supplementary material available at 10.1186/s12870-024-05003-w.

## Introduction

Chinese cabbage is an important vegetable crop in the Brassica family of Cruciferae. It undergoes cross-pollination through bisexual flowers and exhibits significant heterosis. Utilizing a male sterile line serves as an optimal method for hybrid seed production [1, 2]. Understanding the genes and molecular mechanisms associated with male sterility is pivotal for advancing Chinese cabbage hybrid breeding and thus holds substantial economic implications.

Male sterility primarily involves anther development, a delicate and complex process encompassing anther wall formation and pollen grain generation. The anther wall comprises layers such as the epidermis, endoderm, middle layer, and tapetum, progressing from the outer layers to the inner layers. Tapetum cells transform into secretory cells post-formation, while haploid microspores become enveloped by callose. As the anther grows, the microspores released from the tetrads gradually mature into pollen grains within the anther chamber [3–5]. Throughout anther development, processes like tapetum formation and apoptosis, meiosis, callose production and degradation, and pollen wall formation intricately influence pollen development. These tightly regulated processes, if disrupted, lead to varying degrees of reduced male fertility [6].

Long non-coding RNAs (lncRNAs), extending beyond 200 nucleotides, wield significant influence across diverse life processes, modulating crucial biological pathways, including plant flowering, photomorphogenesis, reproduction, and responses to abiotic/biological stress [7–9]. Increasingly, studies highlight the involvement of numerous lncRNAs in male reproductive development in plants. In maize, *Zm401* plays a role in anther development by functioning as short open reading frame mRNA (sORF mRNA) and/or non-coding RNA (ncRNA). Knockout of *Zm401* significantly affects the expression of key pollen development genes (*ZmMADS2*, *Zm3-3*, and *ZmC5*), resulting in abnormal development of microspore and tapetum and ultimately male infertility [10–12]. In rice, the long-day-specific male-fertility-associated RNA (*LDMAR*) has been identified as an lncRNA capable of regulating photosensitive male sterility. Under extended daylight conditions, highly expressed *LDMAR* maintains normal pollen development [13]. Conversely, mutations in ldmar alter the secondary structure of *LDMAR*, causing increased methylation in the *LDMAR* promoter region. This reduction in *LDMAR* transcription levels initiates premature programmed cell death of anther tapetum cells under extended daylight, leading to photosensitive male sterility [14]. Furthermore, the photosensitive sterility gene *PMS1* generates the transcription of lncRNA *PMS1T* in rice. This lncRNA, targeted and activated by miR2118, generates 21-nt phasiRNAs that preferentially accumulate in photosensitive male sterility under prolonged sunlight conditions, actively participating in rice reproductive development [15].

Similarly, several lncRNAs have been implicated in pollen development within Chinese cabbage. LncRNA *BcMF11* is specifically expressed in anthers. Suppression of *BcMF11* results in delayed tapetum degradation, ultimately affecting pollen development [16, 17]. A recent study of Brassica highlighted the expression of 12,051 lncRNAs during pollen development and fertilization [18]. Another investigation identified 4,347 lncRNAs and 2,045 lncNATs across five stages of pollen development, utilizing genome-wide identification methods [19]. The utilization of diverse male sterile materials in identifying lncRNAs might reveal corresponding anther development processes. Moreover, lncRNAs often act as regulators of the transcription factors or protein-coding genes that participate in the formation of male sterility. Comprehensive studies are undoubtedly important for interpreting lncRNA-mediated gene expression networks linked to anther development and male sterility.

In this study, a new Chinese cabbage genic male sterile line (366–2 S) was characterized. During anther development, the tapetum of 366–2 S remained intact for a long time and presented delayed degradation. Additionally, the tetrads of 366–2 S remained unseparated and clustered together, resulting in the absence of pollen release and subsequent male sterility. This study was carried out to identify differentially expressed lncRNAs and mRNAs involved in anther development through lncRNA and mRNA sequencing using fertile line 366–2 F and sterile line 366–2 S. Furthermore, to find out the potential lncRNA-mRNA modules associated with male sterility, target gene prediction analysis of lncRNAs and GO enrichment analysis of mRNAs were performed. This study will broaden our knowledge of the role of lncRNAs and the molecular mechanisms underpinning male sterility in Chinese cabbage.

## Results

### Characterization of abnormal anther development in 366–2 S

**Fig. 1:**
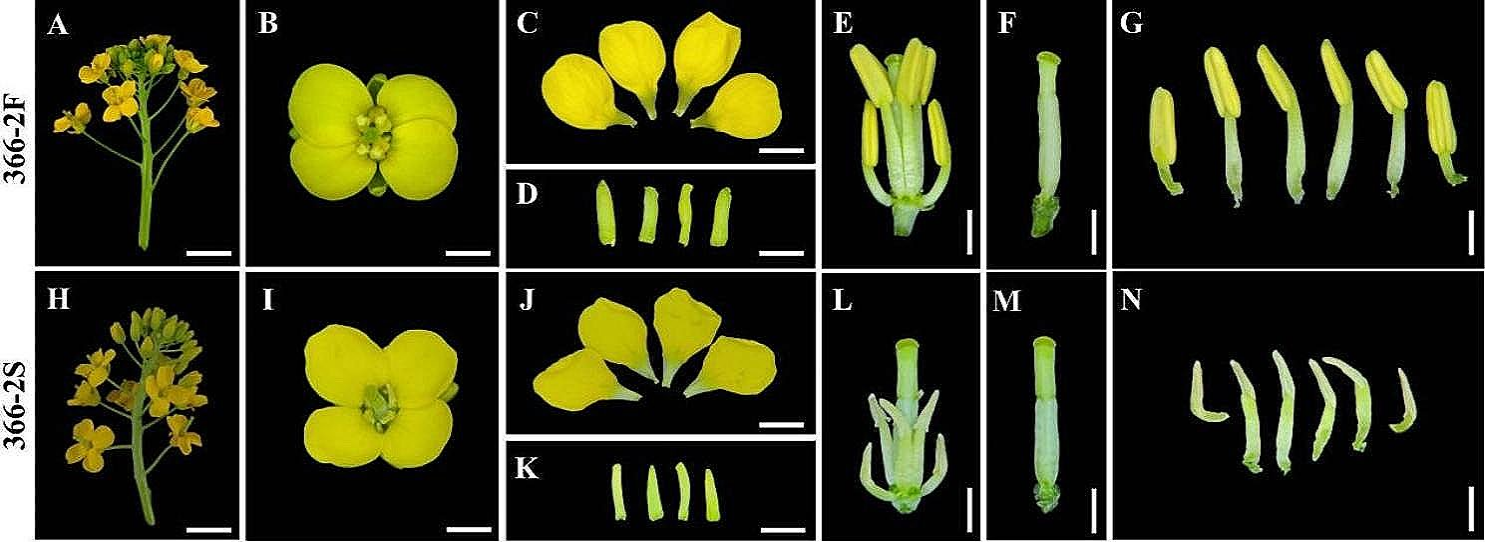
Morphological characteristics of floral organs of Chinese cabbage at full flowering stage of 366–2 S and 366–2 F lines. (**A–G**) A floret at the anthesis stage with normal flower organs in 366–2 F. (**H–N**) A floret at the anthesis stage with shorter filaments and anthers without pollen in 366–2 S. *Bar* = 5 mm

During the pollination phase, the sterile line 366–2 S was distinguished from the progenies of the heterozygous fertile line 366-2. Anther appearance within flowers exhibited notable differences between the 366–2 F and 366–2 S lines (Fig. 1). Both 366–2 F and 366–2 S showed typical flower traits, blooming normally with four petals (Fig. 1, C, J), four sepals (Fig. 1, D, K), and one pistil adhering to six stamens (Fig. 1, E, L). However, distinct variations were observed in the anthers of these lines. In 366–2 F, the anthers appeared yellow and plump (Fig. 1, G), exhibiting normal dehiscence and the release of active pollens. Conversely, the anthers of 366–2 S appeared gray and withered (Fig. 1, N), failing to release pollens, indicating a lack of pollen development within 366–2 S.

**Fig. 2:**
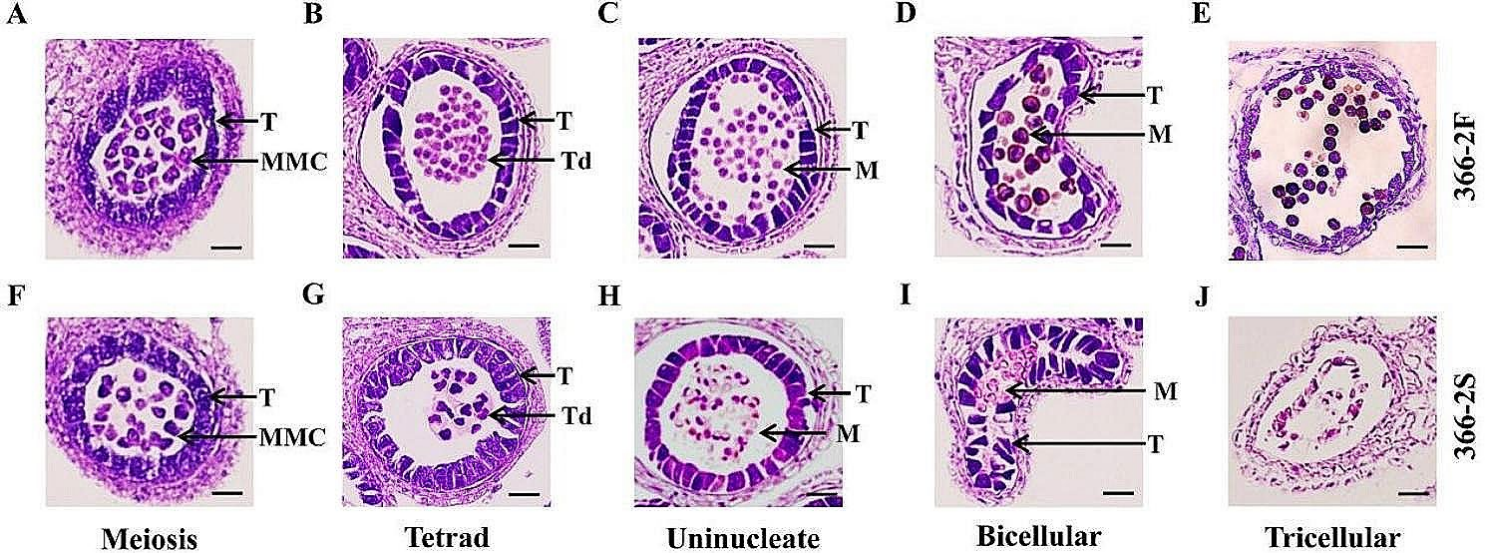
Paraffin sections of Chinese cabbage anthers of 366–2 F and 366–2 S. (**A–E**) 366–2 F pollen microspore development process. (**F–J**) 366–2 S pollen microspore development process; the tetrad period began to develop abnormally. T: Tapetum; MMC: Microspore mother cell; Td: Tetrad; M: Microspore. *Bar* = 20 μm

To understand the abnormal pollen development in 366–2 S, we meticulously observed the anthers at five consecutive developmental stages (meiosis, tetrad, uninucleate, bicellular, and tricellular) utilizing HE staining. At the meiosis and tetrad stages (Fig. 2, A, B, F, G), no obvious differences were discernible between 366 and 2 F and 366–2 S. Both exhibited similar and normal tapetums, microspore mother cells (MMCs), and tetrads. However, notable deviations emerged at the uninucleate and bicellular stages. In 366–2 F, the tetrads dissociated normally, releasing microspores (Fig. 2, C, D). Conversely, in 366–2 S, the tetrads remained unseparated, clustering together (Fig. 2, H, I), resulting in the absence of microspore formation. At the tricellular stage, microspores were spherical, and the anther chambers were filled in 366–2 F (Fig. 2, E). In contrast, anther chambers in 366–2 S showed atrophy, with only a few contents remaining (Fig. 2, J), indicating signs of male sterility. At the bicellular and tricellular stages, the tapetum underwent gradual degradation, ceasing the provision of necessary molecules for microspore development. Nevertheless, the tapetum of 366–2 S appeared more robust compared to that in 366–2 F (Fig. 2). This delayed degradation of the tapetum might contribute to male sterility in 366–2 S.

### Identification of lncRNAs and prediction of their target genes in anthers

**Fig. 3:**
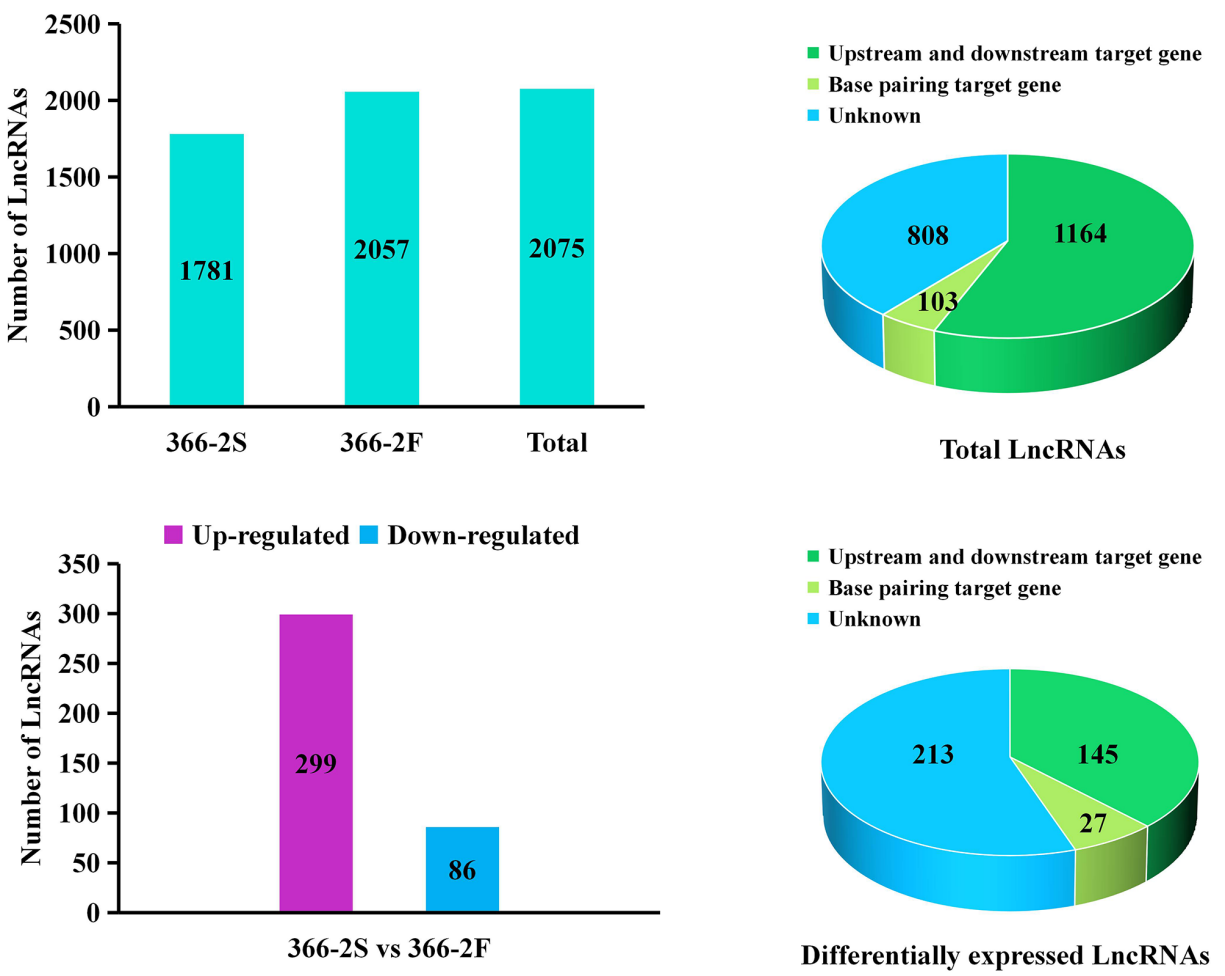
LncRNA sequencing in anthers of 366–2 S and 366–2 F. (**A**) Identification of lncRNAs in 366–2 S and 366–2 F. (**B**) Target gene prediction of lnRNAs. (**C**) Identification of DE-lncRNAs between 366–2 S and 366–2 F. (**D**) Target gene prediction of DE-lncRNAs between 366–2 S and 366–2 F

To analyze the role of lncRNAs in pollen development of both 366–2 S and 366–2 F, a comparative lncRNA-seq was conducted using anthers harvested from unopened buds of varying lengths. A substantial quantity of clean reads were obtained, totaling 92,287,338 for 366–2 S and 92,214,922 for 366–2 F (Table 1). These reads exhibited high quality, surpassing Q20 and Q30 thresholds of over 99%, with mapped rates exceeding 83% (Table 1). By aligning with the reference genome and conducting CPC coding potential prediction, 1,781 lncRNAs were identified in 366–2 S and and 2,057 lncRNAs were identified in 366–2 F. Ultimately, a total of 2 075 lncRNAs were obtained (Fig. 3, A and Supplementary Table S2). The target genes were predicted through upstream and downstream gene sequence alignment and base pairing. Among these, 1,164 lncRNAs had both upstream and downstream target genes, and 103 lncRNAs exhibited base pairing target genes, while 808 lncRNAs had no corresponding target genes (Fig. 3, B and Supplementary Table S2), resulting in the prediction of a total of 2,376 target genes (Supplementary Table S2). Differential expression analysis of lncRNAs between 366 and 2 S and 366–2 F revealed significant differential expression in 385 lncRNAs within 366–2 S, with 299 and 86 showing upregulation and downregulation, respectively (Fig. 3, C and Supplementary Table S2). Correspondingly, the predicted target genes associated with these DE-lncRNAs were identified (Fig. 3, D and Supplementary Table S2). These DE-lncRNAs and their corresponding predicted target genes are implicated in the abnormal pollen development observed in 366–2 S.

**Table 1:** LncRNA sequencing data of sterile and fertile Chinese cabbage lines

Samples	Clean reads	Mapped reads	Mapped ratio (%)	Q20 (%)	Q30 (%)
366–2 S	92,287,338	79,094,621	85.77	99.98	99.54
366–2 F	92,214,922	76,949,037	83.38	99.99	99.62

### Differential expression of mRNAs in anther development

**Fig. 4:**
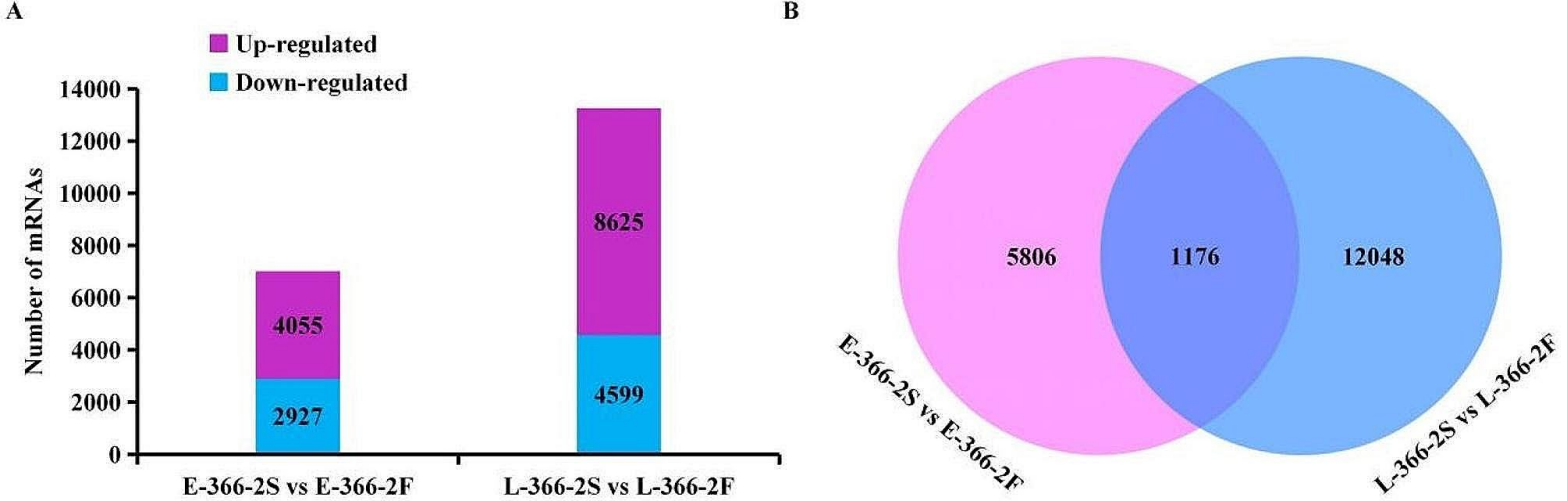
mRNA sequencing in anthers of 366–2 S and 366–2 F. (**A**) Identification of DE-mRNA between 366–2 S and 366–2 F. (**B**) Venn analysis of DE-mRNAs at the early and late stages

The anthers of small buds (about 1 ∼ 1.5 mm) in 366–2 S showed signs of sterility. To understand the differences in mRNA expression in anther development of 366–2 S and 366–2 F, comparative transcriptome analysis was performed at the early-stage (when bud length was approximately 1 ∼ 1.5 mm) and the late-stage (when bud length was approximately 3.5 ∼ 5 mm) of anther development. The sequencing yielded an average of 42,154,243; 41,243,279; 41,333,469 and 41,328,565 clean reads with 85.76%, 86.67%, 88.03%, and 87.86% of reads successfully mapped to the reference genome for E-366–2 S, E-366–2 F, L-366–2 S, and L-366–2 F, respectively (Table 2). A comprehensive total of 40,482 mRNAs were identified and annotated (Supplementary Table S3). The expression levels of mRNAs exhibited a highly significant correlation among the three repetitions in each treatment (Supplementary Fig. S1), affirming the reliability of the sequencing data. Subsequently, 6,982 mRNAs (4,055 upregulated and 2,927 downregulated) were identified in the early group (E-366–2 S vs. E-366–2 F), along with 13,224 mRNAs (8,625 upregulated and 4,599 downregulated) in the late group (L-366–2 S vs. L-366–2 F) (Fig. 4, A). Furthermore, 1,176 consistent mRNAs were identified from the two groups (Fig. 4, B). The observation indicated that the pollen of 366–2 S was abnormal compared to that of 366–2 F at both the early and late stages. Thus, these consistent DE-mRNAs are anticipated to be associated with pollen development and male fertility.

**Table 2:** mRNA sequencing data of 12 samples

Samples	Clean Reads	Mapped Reads	Mapped Ratio (%)	Q30(%)
E-366–2 S-1	40,814,468	34,839,230	85.36	92.54
E-366–2 S-2	43,252,172	37,408,804	86.49	92.38
E-366–2 S-3	42,396,088	36,218,978	85.43	92.48
**E-366–2 S-average**	**42,154,243**	**36,151,479**	**85.76**	**92.47**
E-366–2 F-1	41,267,436	35,667,445	86.43	91.98
E-366–2 F-2	41,220,446	35,964,839	87.25	92.38
E-366–2 F-3	41,241,956	35,608,305	86.34	92.29
**E-366–2 F-average**	**41,243,279**	**35,745,550**	**86.67**	**92.22**
L-366–2 S-1	41,265,346	36,078,292	87.43	92.89
L-366–2 S-2	41,741,410	36,891,058	88.38	92.07
L-366–2 S-3	40,993,652	36,189,196	88.28	92.30
**L-366–2 S-average**	**41,333,469**	**36,385,853**	**88.03**	**92.42**
L-366–2 F-1	43,295,808	37,927,128	87.6	92.45
L-366–2 F-2	40,589,994	35,633,956	87.79	92.60
L-366–2 F-3	40,099,894	35,348,057	88.15	93.65
**L-366–2 F-average**	**41,328,565**	**36,311,277**	**87.86**	**92.90**

### Go enrichment analysis of DE-mRNAs

**Fig. 5:**
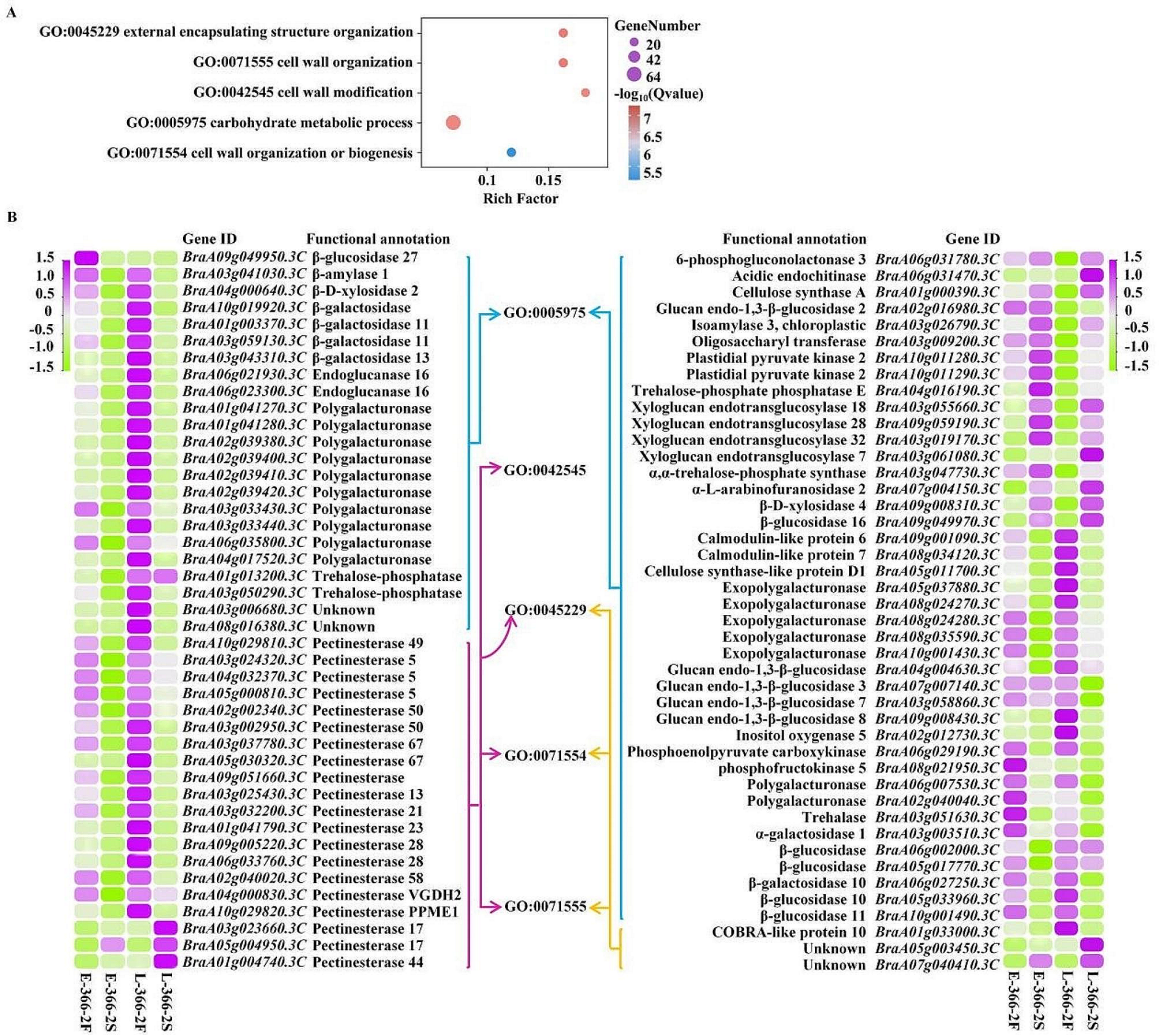
GO enrichment analysis of differentially expressed mRNAs. (**A**) Five GO terms associated with the anther cell wall were significantly enriched. (**B**) A total of 87 DEGs were contained in the significantly enriched GO.terms

Based on the BP, GO enrichment analysis was performed for fertility-related DE-mRNAs. Five GO terms, i.e., GO: 0045229 (external encapsulating structure organization), GO: 0071555 (cell wall organization), GO: 0042545 (cell wall modification), GO: 0005975 (carbohydrate metabolic process), and GO: 0071554 (cell wall organization or biogenesis), were significantly enriched (Fig. 5, A, Supplementary Table S4). Significantly enriched GO terms contained a total of 87 DE-mRNAs (Fig. 5, B). Among them, in GO: 0045229, GO: 0071555, GO: 0042545, and GO: 0071554, 20 mRNAs encode pectinesterases; in GO: 0005975, 17 mRNAs encode polygalacturonases, all of which belong to pectinase regulating the degradation of pectins in the cell wall. In addition, many other mRNAs in GO: 0005975 encode some glycosidases, such as galactosidase (*BraA03g003510.3 C*, *BraA06g027250.3 C*, *BraA01g003370.3 C*, *BraA03g059130.3 C*, *BraA03g059130.3 C*, and *BraA10g019920.3 C*) and glucosidase (*BraA02g016980.3 C*, *BraA02g016980.3 C*, *BraA02g016980.3 C*, *BraA02g016980.3 C*, *BraA02g016980.3 C*, *BraA05g017770.3 C*, *BraA06g002000.3 C*, *BraA05g033960.3 C*, *BraA05g033960.3 C*, *BraA09g049970.3 C*, and *BraA09g049950.3 C*). They might be involved in the hydrolysis of polysaccharides in the cell wall. Notably, most of these mRNAs showed a downregulation pattern in 366–2 S (Fig. 5, B), which potentially impaired the ability of cell wall degradation.

### Major lncRNA-mRNA modules associated with pollen fertility

**Fig. 6:**
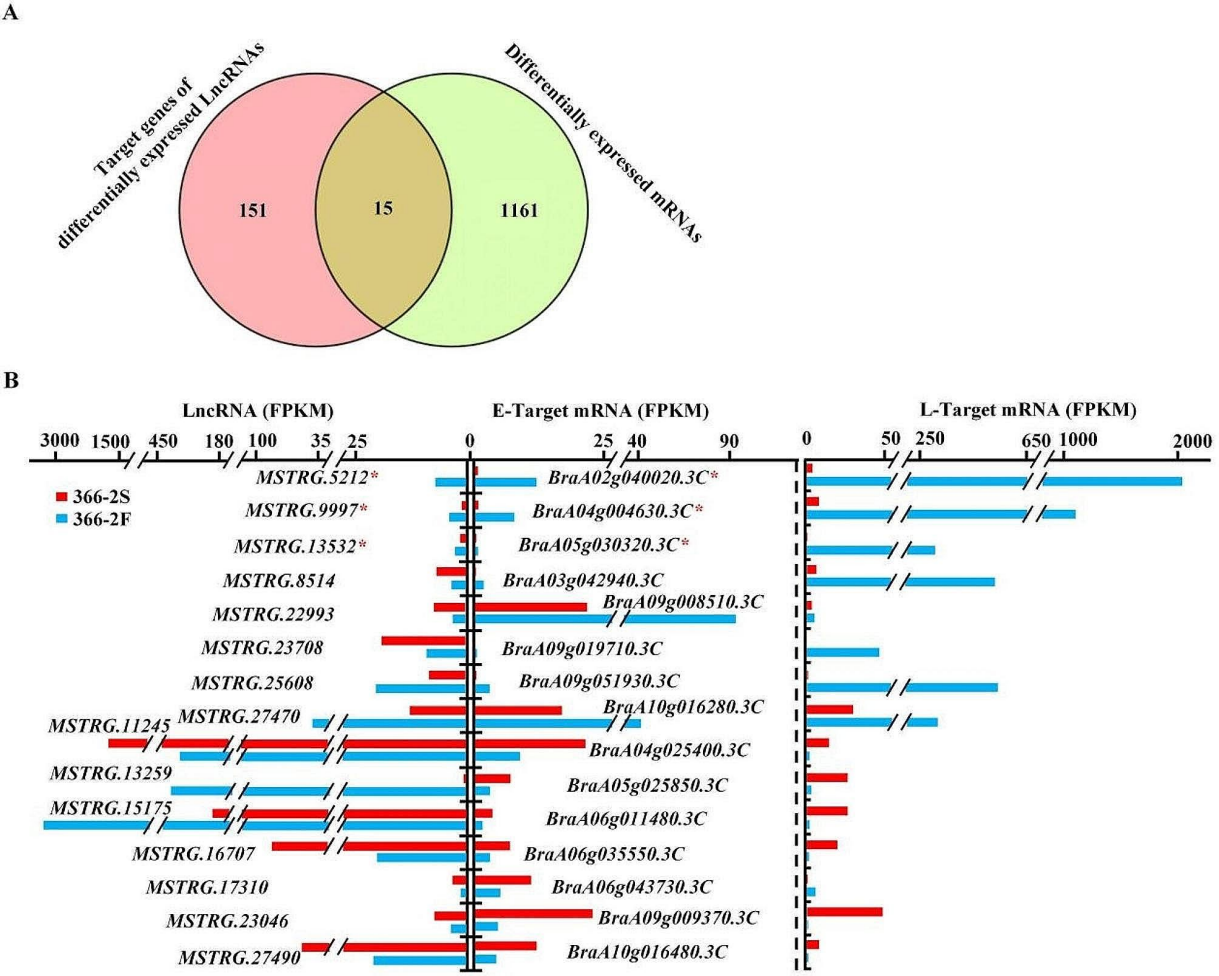
Identification and analysis of differential lncRNAs and target mRNAs. (**A**) A total of 15 lncRNA-mRNA modules were identified. (**B**) FPKM expression levels and corresponding relationships of lncRNAs and mRNAs in the identified module. * indicates that mRNAs belong to significantly enriched GO terms

The role of lncRNAs in regulating the expression of target genes is pivotal in various BPs. A Venn diagram (Fig. 6, A, Supplementary Table S5) revealed 15 DE-mRNAs that corresponded to the target genes of DE-lncRNAs. Among these, certain lncRNAs exhibited consistent expression trends with their target mRNAs, exemplified by *MSTRG.9997*-*BraA04g004630.3 C*; while others showed opposing trends, such as *MSTRG.8514*-*BraA03g042940.3 C* (Fig. 6, B). This indicates the potential of lncRNAs to either activate or inhibit their gene expressions. Notably, three target mRNAs, namely, *BraA04g004630.3 C* (β-1,3-glucanase), *BraA02g040020.3 C* (pectinesterase), and *BraA05g030320.3 C* (pectinesterase), belonged to the significantly enriched GO terms as previously identified (Fig. 6, B). They were downregulated along with their corresponding lncRNAs in 366–2 S. Therefore, the *MSTRG.9997-BraA04g004630.3 C*, *MSTRG.5212-BraA02g040020.3 C*, and *MSTRG.13,532-BraA05g030320.3 C* were considered the major lncRNA-mRNA modules associated with pollen fertility/sterility.

### Validation of lncRNA and mRNA expression by qPCR

**Fig. 7:**
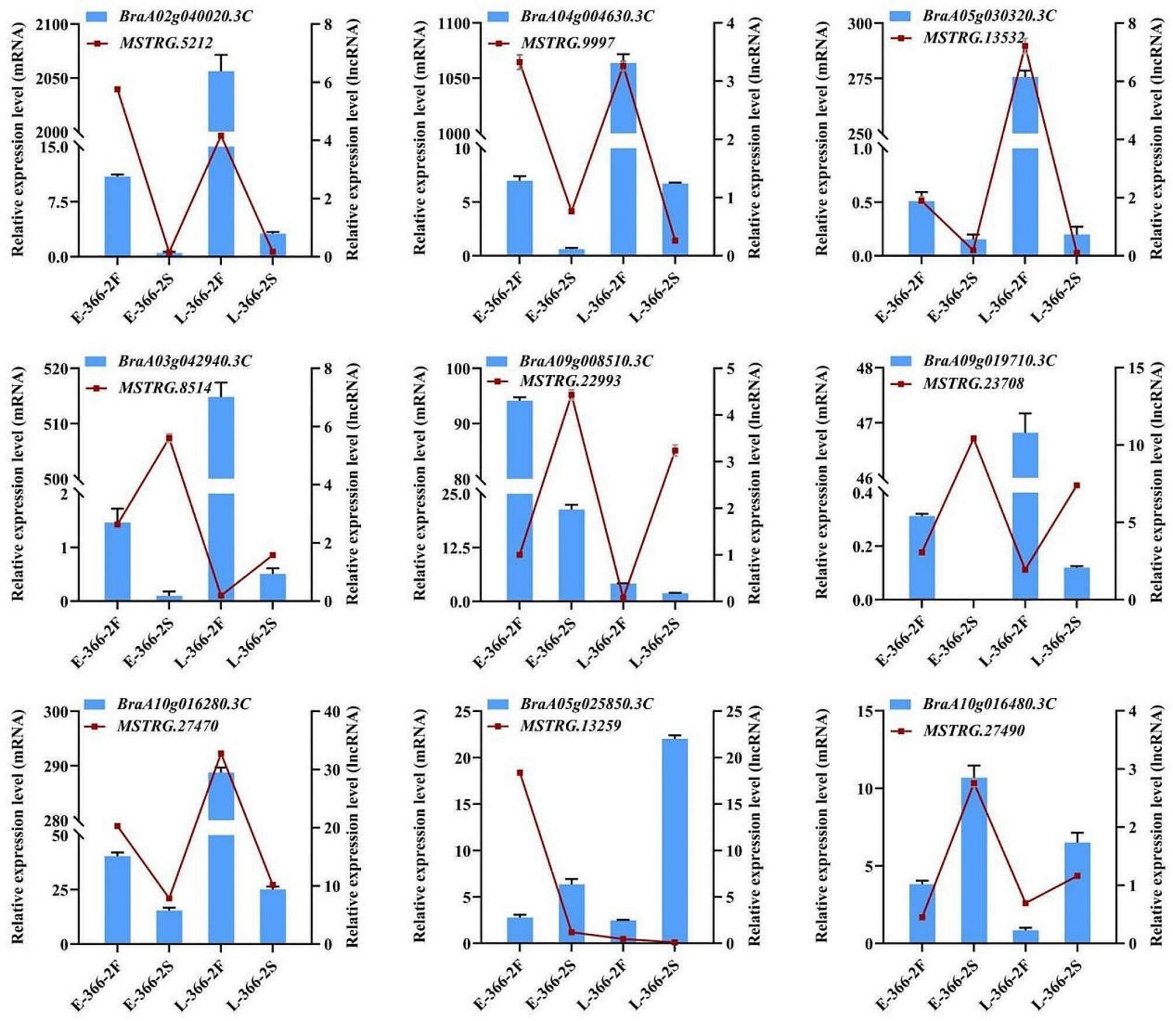
Expression validation of mRNAs and lncRNAs by qPCR. The relative expression of E-366–2 F was used as the control in qPCR analysis

To verify the accuracy of the lncRNA-seq and mRNA-seq results, the relative expression levels of nine lncRNA-mRNA modules were investigated by qPCR. The expression patterns of most selected lncRNAs and genes analyzed by qPCR were similar to the expression levels that were computed using sequencing data (Fig. 7). Although the magnitude of changes in the expression level of the selected lncRNAs and mRNAs varies, the expression trends were consistent with expectations in sequencing, indicating that the sequencing data were reliable.

## Discussion

### Abnormalities of the tapetum resulted in the male sterility of 366–2 S

From the formation of sporogenous cells, pollen mother cells are produced by mitosis; then, they form tetrads by meiosis. After the tetrads are dissociated, microspores are released and gradually develop into pollen grains [20]. The abnormality of these processes will lead to pollen abortion or a total absence of pollens [21]. In this study, Chinese cabbage mutant 366–2 S indicated abnormal anthers and pollens, which are typical characteristics of male sterility. Compared with the wild type 366–2 F, tetrads of 366–2 S could not be dissociated or they could not release microspores, which were directly responsible for male sterility. The degradation of tetrads depends on the callase enzyme (β-1,3-glucanase), which typically hydrolyzes the callose wall encompassing four microspores in a tetrad to promote the release of microspores [22]. Many studies have shown that β-1,3-glucanase is secreted by the tapetum cells [23–26]. Some abnormalities in the tapetum cells may affect the secretion or the function of β-1,3-glucanase and thus lead to the occurrence of male sterility. Cytological observations of anther development also showed that the tapetum in 366–2 S remains intact for a long time and presents delayed degradation. Therefore, these results suggested that some abnormalities of the tapetum delay self-degradation and subsequently affect the action of β-1,3-glucanase on dissociating the tetrads and releasing the microspores, which brings about male sterility of 366–2 S.

### Differentially expressed lncRNAs between 366 and 2 F and 366–2 S associated with male sterility

Several lncRNAs have been identified and cloned, and they have been proven to be involved in anther development in different plants, such as maize *(Zm401*) [10–12], rice (*LDMAR* [13], *PMS1T* [14, 15], and *eTM160* [27]), Brassica campestris (*BcMF11)* [16, 17], and Brassica rapa (*eTM160*) [18]. Moreover, it was found that these lncRNAs play a dominant role in a specific stage, such as the microspore mother cell stage, tetrad stage, mononuclear stage, and mature pollen stage, corresponding to the male sterile characteristics of their respective sterile lines. Thus, the use of different male sterile materials is conducive to the identification of new lncRNAs with male sterility. Huang et al. (2018) used the Chinese cabbage genic male sterility (GMS) A/B line (‘Bcajh97-01 A/B’) for lncRNA sequencing at five pollen developmental stages and identified 2,320 and 1,693 lncRNAs specifically expressed in fertile or sterile floral buds, respectively. Through the target gene prediction of lncRNAs, many pollen development-related genes were matched, most of which had been identified as male sterility genes. Thus, these results preliminarily provided some lncRNA databases associated with male sterility in Chinese cabbage. In this study, the Chinese cabbage fertile/sterile lines (366–2 F/S) were used for analyzing the roles of lncRNAs in pollen development. A total of 385 DE-lncRNAs were identified in 366–2 S compared with 366–2 F. The male sterility of 366–2 S primarily resulted from the delayed degradation of the tapetum and the non-separation of tetrads. Conversely, in the sterile line Bcajh97-01 A, disruptions occurred during the meiosis of the pollen mother cells, leading to the inability of the anther to form tetrads [28]. The use of different male sterile materials for identifying lncRNAs may provide insights into distinct developmental processes of pollen. In addition, compared to previously validated lncRNAs, a homology comparison revealed that none of them were identified both in 366–2 F and 366–2 S, which might be due to the different sampling stages or methods or due to the low expression level. Therefore, the DE-lncRNAs identified in this study were potentially different and would enrich lncRNA datasets associated with male sterility in Chinese cabbage.

### A large number of downregulated mRNAs related to cell wall degradation potentially delayed the degradation of the tapetum

In this study, a total of 1,176 DE-mRNAs were screened by comparative transcriptome analysis of anthers between 366 and 2 S and 366–2 F. GO enrichment analysis showed that carbohydrate metabolic and cell wall-related processes, such as GO: 0071555 and GO: 0005975, are significantly enriched. The mRNAs involved in these processes encode pectinesterases, polygalacturonases, galactosidase, and glucosidase, all of which are involved in cell wall degradation. Most of these mRNAs were downregulated in 366–2 S, potentially inhibiting cell wall degradation during certain developmental processes. Coincidentally, delayed degradation of the tapetum was observed in the cell sections of 366–2 S. The cytological features of tapetal degradation primarily include the shrinkage of the cell, the rupture of the liquid bubble, the cavity of the cell, and the cracking of the cell wall [29]. Tapetal cells encircle the innermost anther wall layer (named tapetum), which surrounds the male reproductive cells, and undergo programmed cell death-triggered degradation after the meiosis of pollen mother cells [30, 31]. Tapetal degradation is essential for healthy pollen development to provide many molecules including nutrients, proteins, lipids, and polysaccharides; as a result, premature or delayed tapetal degradation causes male sterility [32]. Therefore, we concluded that these downregulated mRNAs related to cell wall degradation potentially hinder tapetal degradation, which then causes male sterility in 366–2 S.

### Candidate lncRNA-mRNA regulated the pollen development in Chinese cabbage

LncRNAs target the mRNAs as lncRNA-mRNA modules to play a role in multiple processes of growth and development. For example, lncRNA *HID1*, which is associated with photomorphogenesis, can assemble into large nuclear protein-RNA complex(es) and bind to Phytochrome Interacting Factor 3 (PIF3) to inhibit its transcription, thereby regulating the development of Arabidopsis hypocotyl [33]. In this study, three major lncRNA-mRNA modules were identified, including one module (*MSTRG.9997-BraA04g004630.3 C*) associated with callose degradation and two modules (*MSTRG.5212-BraA02g040020.3 C* and *MSTRG.13,532-BraA05g030320.3 C*) associated with cell wall degradation, which might contribute to the male sterility of 366–2 S. Indeed, *BraA04g004630.3 C* encodes a β-1,3-glucanase, which can specifically catalyze the hydrolysis of callose wall during pollen development. In rice, the silencing of *Osg1*, a β-1,3-glucanase gene, resulted in the disrupted callose degradation in the tetrads and delayed release of young microspores and caused male sterility [34]; the scenario is similar to the results of our study. In 366–2 S, *MSTRG.9997* was downregulated and then targeted *BraA04g004630.3 C* to bring about a low level of β-1,3-glucanase, probably causing non-separation of tetrads and non-production of microspores. *BraA02g040020.3 C* and *BraA05g030320.3 C* encode pectinesterase, by which pectin can be demethylated and then cleaved by endo-polygalacturonases, resulting in cell wall loosening. Many studies found that pectinesterase plays an important role in pollen and pollen tube development and its functional deficiency may lead to male sterility [35–37]. Thus, it seems that these two lncRNA-mRNA modules might not be causal factors but downstream results along with the male sterility occurrence in 366–2 S. However, cytological observations showed that tapetum in 366–2 S presents delayed degradation, which must involve the process of cell wall degradation. Probably, certain pectinesterases (such as *BraA02g040020.3 C* and *BraA05g030320.3 C*) also played a role in the degradation of tapetum, and these two lncRNA-mRNA modules had some key downstream links. Therefore, these three candidate lncRNA-mRNA modules might potentially be involved in regulating the pollen development in Chinese cabbage.

## Conclusions

This study revealed that the abnormal pollen development in the Chinese cabbage sterile line 366–2 S might start at the tetrad stage, including the delayed degradation of the tapetum and the non-separation of tetrads. Through lncRNA sequencing, 385 DE-lncRNAs were identified between 366 and 2 F and 366–2 S, which are probably related to male sterility of 366–2 S. To understand exactly the alterations in mRNA expression and identify the potential target genes of lncRNAs, comparative transcriptome analyses were performed at two pollen developmental stages. A total of 1,176 DE-mRNAs were screened. GO enrichment analysis showed that carbohydrate metabolic and cell wall-related processes are significantly enriched, involving a large number of downregulated mRNAs related to cell wall degradation, which potentially play a role in delaying the tapetal degradation. Furthermore, a total of 15 lncRNA-mRNA modules were identified. Among them, three major lncRNA-mRNA modules were considered to be closely related to the male sterility of 366–2 S. Among them, *MSTRG.9997-BraA04g004630.3 C* (β-1,3-glucanase) was probably associated with callose degradation and separation of tetrads; *MSTRG.5212-BraA02g040020.3 C* (pectinesterase) and *MSTRG.13,532-BraA05g030320.3 C* (pectinesterase) were probably associated with cell wall degradation of tapetums, which is typically involved in regulating pollen development. Overall, this study provides some important clues and evidence that lncRNAs participate in pollen development, and it also lays the basic genomic foundation for further investigations.

## Methods

### Plant materials and cultivation

The genic male sterile mutant line 366–2 S originated as a natural mutant derived from the Chinese cabbage breeding material 366-2 population, sourced from the National Vegetable Germplasm Resource Intermediate Bank, Institute of Vegetable and Flower, Chinese Academy of Agricultural Sciences. The homozygous fertile line was identified as the wild type (366–2 F). Upon crossing 366–2 S with 366–2 F, the resulting F_1_ population presented a fertile type, while the F_2_ population exhibited a segregation ratio of fertile to sterile types at 3:1 due to a recessive single gene mutation present in 366–2 S. Additionally, in contrast with the tapetum of the fertile 366–2 F, the tapetum observed in the sterile 366–2 S consistently persisted without undergoing degradation, presenting a notable deviation from typical male sterile mutants. The cultivation of all plant materials took place in the standardized experimental field situated at the Henan Modern Agriculture Research and Development Base in Yuanyang, Henan Province, China.

### Cytological observation

For identifying the period of pollen abortion, the flower buds of 366–2 S and 366–2 F were fixed by using FAA solution (50% ethanol, 5% glacial acetic acid, and 10% formalin) and then dehydrated in a graded ethanol series. The prepared anthers were embedded in paraffin and stained with hematoxylin and eosin and observed under an optical microscope (Nikon ECLIPSE 80i; Nikon, Japan). The buds were subsequently divided into five groups considering the length of the buds (BUD1 < 1 mm, BUD2: 1 ∼ 1.5 mm, BUD3: 1.5 ∼ 2.5 mm, BUD4: 2.5 ∼ 3.5 mm, and BUD5 > 3.5 mm).

### Sample preparation and transcriptome sequencing

A single biological replicate was prepared for lncRNA sequencing, aiming for accuracy and sequencing efficacy. Anthers from the 366–2 S line were combined from multiple buds of different lengths (ranging between 1 and 5 mm) before flowering, ensuring a diverse representation. Similar types of anthers from the 366–2 F line were utilized as the control group. The extraction of total RNA was conducted using the TRIzol reagent method (Invitrogen, Carlsbad, CA, USA). The concentration and quality of RNA were evaluated by using a NanoDrop 2000 system (Thermo Fisher Scientific, Wilmington, DE) and an Agilent Bioanalyzer 2100 system (Agilent Technologies, CA, USA). Ribosomal RNA (rRNA) and poly-A enriched RNA (most coding sequences) were removed from the total RNA. The remaining RNA was randomly fragmented into short fragments for constructing libraries of sequencing. First-strand cDNA was synthesized by using random hexamers and M-MuLV reverse transcriptase; second-strand cDNA was subsequently synthesized by using DNA polymerase I and RNase H; after using AMPure XP beads to purify double-stranded cDNA, they were subjected to end repair, addition of poly-A tail, ligation of sequencing linker, and fragment size selection. Finally, two cDNA libraries were subjected to PCR experiments and sequenced in Illumina HiSeq 2500 platform (150 bp paired-end sequencing; 15 Gb data size).

Anthers of parallel buds at the early stage (E; about 1 ∼ 1.5 mm) and parallel buds at the late stage (L; about 3.5 ∼ 5 mm) were selected for mRNA sequencing. In total, 12 samples including four treatments (E-366–2 S, E-366–2 F, L-366–2 S, and L-366–2 F) with three replicates were used. The mRNA (coding sequences) was enriched with a poly-A tail from total RNA by using magnetic beads with Oligo dT. Subsequently, the sequencing libraries of all samples were constructed. Finally, 12 cDNA libraries were sequenced in the Illumina HiSeq 2500 platform (150 bp paired-end sequencing; 6 Gb data size).

### LncRNA identification and target gene prediction

FastQC software [38] was used to confirm the quality (Q20, Q30) of lncRNA-seq data. Clean reads were obtained by removing reads with an adapter and containing poly-N (an ambiguous sequence content exceeding 10%) and low-quality reads (Q20 < 80%). After aligning the clean reads to the *B. rapa* reference genome (V3.0) [39], using HISAT2 software [40], all mapped reads were assembled into transcripts by using the StringTie software [41]. Considering the incomplete transcript assembly due to gaps in coverage, artificial reads were constructed and used for assembly based on reference transcripts. The transcripts with lengths less than 200 nt or possessing protein-coding ability were filtered out. Furthermore, the protein-coding potential of the remaining transcripts was predicted by using the CPC software [42]. The transcripts without protein-coding ability were identified as lncRNAs.

Given the small sample size of sequencing, the trans-regulation of lncRNAs was not effectively analyzed. Thus, the target gene prediction of lncRNAs was carried out by co-located and base pairing analysis. An lncRNA located within 10 kb upstream or downstream of a coding gene was considered as co-location genes, and these genes were predicted as the lncRNA’s co-located target genes. The co-located target genes were identified by custom Perl scripts. In addition, the RNAplex package [43] was used to predict the complementary binding between lncRNA and mRNA (coding gene) to obtain the base pairing target genes.

### Differential expression analysis of lncRNAs

Cufflinks software [43] was used to calculate the fragments per kilobase per million (FPKM) value of exons as the expression level of each lncRNA. Differential expression analysis of lncRNAs was performed between 366 and 2 S and 366–2 F (as control) by using the R package (DESeq) [44]. The differentially expressed lncRNAs (DE-lncRNAs) were screened out with a |fold change| ≥ 2 and *p* < 0.01. Further, their corresponding predicted target genes were acquired.

### Identification and differential analysis of mRNA expression profiles

For mRNA-seq data, clean reads from all treatments (E-366–2 S, E-366–2 F, L-366–2 S, and L-366–2 F) were obtained and aligned to the *B. rapa* reference genome (v3.0). Based on the known genome annotation information, the mapped reads were assembled into annotated mRNAs and unannotated mRNAs (potentially representing novel genes). The BLAST software [45] was used to obtain the annotation information for these new genes through sequence alignment with NR (NCBI non-redundant protein sequences) and Gene Ontology (GO) databases. FPKM values were computed for all assembled mRNAs within each sample. Correlation analysis across three replicates for each treatment was performed to assess repeatability. The average of these three replicates was used as the expression profile for each treatment. Differential expression analysis of mRNA profiles was performed within two distinct groups: the early-stage group (E-366–2 S vs. E-366–2 F) and the late-stage group (L-366–2 S vs. L-366–2 F). Differentially expressed mRNAs (DE-mRNAs) were identified for each group using the criteria of |fold change| ≥ 2 and *p* < 0.01. Moreover, overlapped DE-mRNAs between the two groups were determined using a Venn diagram approach to explore commonalities and distinctions in gene expression patterns.

### Go enrichment analysis

Overlapping DE-mRNAs underwent GO enrichment analysis using Blast2GO software [46]. A hypergeometric test served to pinpoint significantly enriched GO terms (biological process, BP). A significance level of 0.01 was selected for the enrichment analysis (*p* < 0.01). In addition, the analysis employed a rich factor to quantify the degree of enrichment. The rich factor gauges the ratio between the proportion of GO terms annotated within the overlapped DE-mRNAs and those present within the annotated mRNAs.

### Screening of lncRNA-mRNA modules associated with male sterility

Some target genes of DE-lncRNAs associated with male sterility were predicted as above. Moreover, the overlapped DE-mRNAs associated with male sterility were identified as above. Through Venn analysis, the consistent mRNAs were selected between these predicted target genes and these overlapped DE-mRNAs. The consistent DE-mRNAs, particularly those associated with significantly enriched GO terms, were potentially paired with corresponding DE-lncRNAs, forming potential lncRNA-mRNA modules that could work together in regulating anther fertility.

### Quantitative real-time PCR (qPCR) analysis

All 12 anther samples from the two stages were re-prepared for a quantitative real-time PCR (qPCR) analysis. Total RNAs from all samples were extracted and reverse transcribed into cDNA using the TransScript one-step gDNA removal and cDNA Synthesis Kit (Trans, Beijing, China). A subset of potential lncRNA-mRNA modules was selected for verification via qPCR. The qPCR experiments were performed using the Roche Light Cycler 480-II system (Roche Applied Sciences, Beijing, China) following specific thermal profiles, with the *BrGAPDH* gene serving as an endogenous control. Each plate included three technical replicates. Specific primers for the selected lncRNAs and mRNAs were designed online via the NCBI website (https://www.ncbi.nlm.nih.gov/) (Supplementary Table S1).

For both lncRNAs and mRNAs, qPCR was performed using TB Green Premix Ex Taq II (Takara, Beijing, China) within a final qPCR reaction mixture containing 5.0 µL of 2×TB Green Premix Ex Taq, 0.8 µL of forward primers, 0.8 µL of reverse primers, 1.2 µL of cDNA, and 2.2 µL of ddH_2_O. Expression levels were determined using the 2^−ΔΔCT^ method [47], with the *BrGAPDH* gene as the reference gene and internal control.

### Electronic supplementary material

Below is the link to the electronic supplementary material.

Supplementary Material 1: Fig. S1 Expression correlation heat map of samples.

Supplementary Material 2: Table S1 The information of the primers used in q-PCR. Table S2 The analysis of lncRNA sequencing. Table S3 The mRNA sequencing analysis. Table S4 GO enrichment analysis of differentially expressed mRNAs based on biological process.

Supplementary Material 3

## Data Availability

The original contributions presented in the study are publicly available. This data can be found here: China National GeneBank DataBase (CNGBdb) under accession number CNP0004830.
