# Supplementary figures and images for: Transcriptome analysis reveals the potential lncRNA-mRNA modules involved in genetic male sterility and fertility of Chinese cabbage (brassica rapa L. ssp. pekinensis)
Source: BMC Plant Biol. 2024 Apr 16;24:289. doi: 10.1186/s12870-024-05003-w (PMC11020818; doi:10.1186/s12870-024-05003-w)

**Supplementary Material-figures**

**
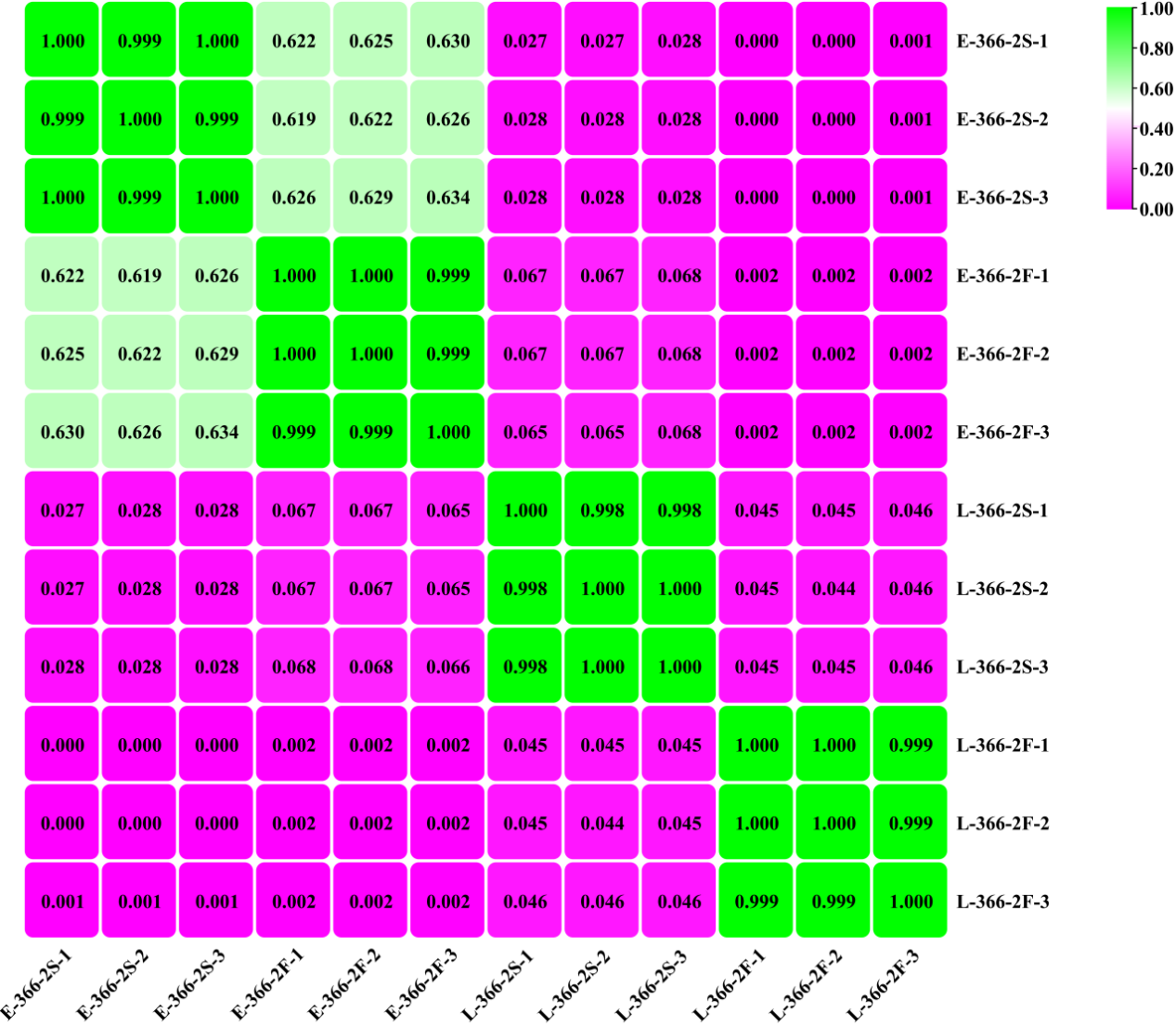
**

**Fig. S1** Expression correlation heat map of samples.

Supplement: Supplementary file 1 — Supplementary Material 1: Fig. S1 Expression correlation heat map of samples. [file 12870_2024_5003_MOESM1_ESM.docx]

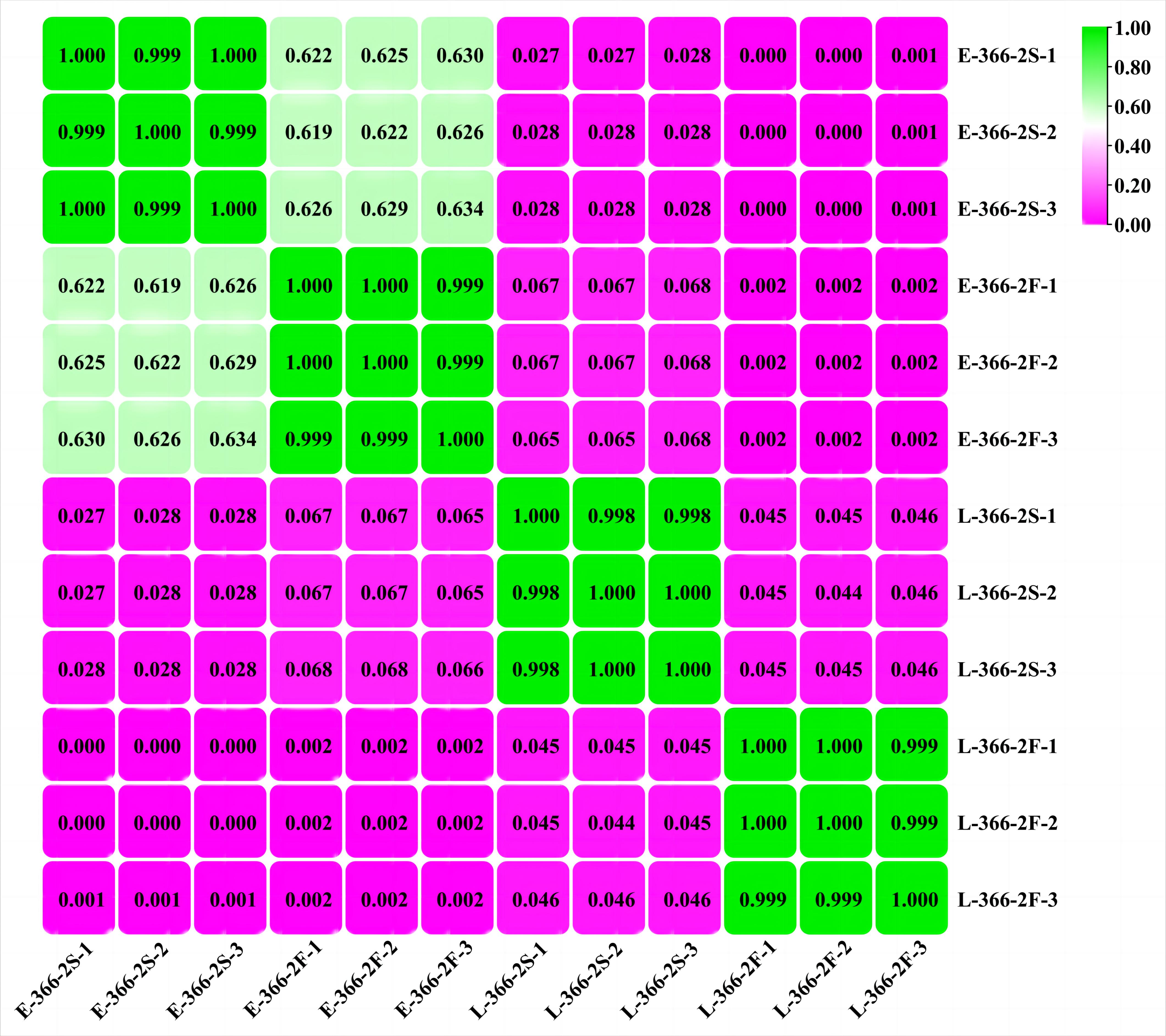

Supplement: Supplementary file 3 — Supplementary Material 3 [file 12870_2024_5003_MOESM3_ESM.jpg]
